# Psychosocial Outcomes of Age Integration Status: Do Age-Integrated Social Networks Benefit Older Adults?

**DOI:** 10.3390/ijerph191912322

**Published:** 2022-09-28

**Authors:** Carly Roman, Christopher R. Beam, Elizabeth Zelinski

**Affiliations:** 1 Leonard Davis School of Gerontology, University of Southern California, Los Angeles, CA 90089, USA; 2 Department of Psychology, University of Southern California, Los Angeles, CA 90089, USA

**Keywords:** age integration, psychosocial well-being, well-being, social engagement

## Abstract

Increased longevity means that older adults have more opportunities to have age-integrated social networks, which include both same-aged peers and intergenerational social ties. Compared to those with peer-only, or intergenerational-only social networks, those with age-integrated networks may experience greater psychosocial benefits due to the age-diverse nature of their social networks. Data from the National Health and Aging Trends Study was used to examine age integration status associations with well-being and social engagement in a nationally representative sample of Medicare beneficiaries in the United States. We hypothesized that age-integrated older adults have greater well-being and social engagement than older adults with peer-only or intergenerational-only networks. Weighted ordinary least squares regression analyses were conducted to test associations of well-being and social engagement with age integration status, controlling for sociodemographic and health covariates. Older adults with age-integrated social networks did not differ in well-being from those with peer-only or intergenerational-only networks, although they had greater social engagement than those with intergenerational-only networks.

## 1. Introduction

Social connections have long been considered an important feature of healthy aging. According to Social Convoy Theory, social networks consisting of emotionally close others are dynamic, changing in structure and function in relation to life events and individual characteristics, such as age, gender, race, health, and marital status [1]. It is well-documented that social connections confer mental and physical health benefits by providing opportunities for older adults to remain mentally and physically active, to fulfill social roles and responsibilities that bolster self-worth, and to provide and receive support [2,3,4]. Although researchers have focused on how social network characteristics are associated with positive psychosocial outcomes such as well-being and social engagement, there are less-considered features that may uniquely contribute to these outcomes. Specifically, intergenerational relationships have not be considered as often in the social network literature but may contribute to psychological health and social engagement.

The current study offers an alternative perspective on how social networks benefit older adults by examining a novel social network feature: age integration status. By definition, age-integrated social networks include same-aged peers and intergenerational connections, distinguishing them from intergenerational-only and peer-only networks. Exposure to individuals not only in one’s own age group, but also in younger generations may provide unique opportunities for older adults to engage in more diverse social settings and become more socially integrated [5,6]. High social integration reflects diverse social connections and diverse social activities [1,7,8,9]. Although social integration literature does not typically focus specifically on age integration, the few studies that have investigated age integration in social networks [10,11,12] are limited by only considering presence of intergenerational social network members without regard for same-aged peers. It is possible that the psychosocial benefits of social integration [1,7,8,13] are similarly experienced by those who are age-integrated, as they too have varied social ties and experiences via their intergenerational connections and same-aged peers. This is supported by research on intergenerational programs that found unique psychosocial benefits for older adults with program-based intergenerational interactions [14,15]. Accordingly, we propose that age integration status is related to social engagement and well-being, with age-integrated older adults having greater well-being and participating in more social activities than older adults with peer-only or intergenerational-only networks.

### 1.1. Social Networks, Well-Being, and Social Engagement

Social networks refer to the social relationships that individuals have, including objective features, like the number of social ties, and subjective features, like the quality of social connections [4,16]. Life events (e.g., getting divorced, becoming a parent) and individual characteristics (e.g., age and gender) shape social convoy features—including whether social network members consist of intergenerational connections or same-aged peers only—in ways that influence well-being and social engagement [1,8,17]. Same-aged peer relationships provide opportunities for companionship, sociability, and social engagement to reinforce meaningful social roles [4,18]; while intergenerational social ties allow older adults to fulfill different types of social roles, in which they express generativity, or care and concern toward younger generations, and experience a sense of purpose and social belonging [4,12,18,19]. The mechanisms through which intergenerational connections and same-aged peers benefit older adults may be distinct, as previously described, or shared, as both types of relationships allow older adults to receive and provide support, possibly through social roles that boost self-esteem [4]. Just as those with high social integration [1,7] and diverse social networks [8,13] have greater well-being and social engagement than those who are less socially integrated and more limited in types of social ties, it is likely that those experiencing age integration in their social networks may reap similar benefits, as the different types of social ties provide more opportunities for and potential pathways through which social connections can positively influence older adults’ well-being and social engagement [4].

The primary pathways through which social networks influence health outcomes include social support, social influence, social engagement and attachment, and access to resources and material goods [4]. For example, social ties may influence older adults to maintain healthy lifestyles by encouraging exercise and abstention from substances, which in turn, positively influences physical health and longevity [3,4]. These factors interact with each other in complex ways (e.g., exercising may support health and well-being that makes it possible for older adults to socially engage with others, while social engagement may provide physical and mental stimulation that allows older adults to exercise).

Empirically, social connections have been associated with positive health outcomes, including reduced mortality [4]. Berkman and Syme [20] found that those with greater social integration, as measured by marital status, familial and non-familial contact, and religious and organizational memberships, had lower mortality rates nearly a decade later compared to those with fewer social ties. Several reviews have confirmed these findings [16,21], highlighting the range of social network measures associated with reduced mortality risk, including simple, binary indicators of social contact (e.g., married or not, living alone or not) and a combination of social network features (e.g., quality and quantity of social ties). These findings extend to mental health, with more socially integrated individuals reporting better mental health and greater social engagement [3,22,23]. Older adults with richer social networks, as measured by number of social network members, number of social network members with frequent contact with very close emotional ties, and number of relationships within different categories (i.e., friend, child, spouse), had better mental health than less socially connected older adults [24]. The authors suggested that poorer mental health has a negative impact on social ties through reinforcement of negative social interactions that make individuals withdraw from social networks; this process is a vicious cycle in which lack of social connectedness further diminishes mental health [25]. On the other hand, stronger social connections reinforce feelings of connectedness that encourage positive psychological changes and further engagement with social ties that bolster well-being. In addition, social connections have been shown to protect against physical [26] and cognitive decline [27,28] over time.

Several theories, including Activity Theory [29] and Successful Aging [30], emphasize that continued engagement in interpersonal and productive activities is necessary for older adults to thrive, psychologically and physically. Although older adults may withdraw from social connections and activities for a variety of reasons, including health issues, loss of friends and family, retirement, or a conscious decision to disengage, these theories posit that participation in social activities is a way for older adults to maintain well-being though social interactions and reinforcement of self-worth [29]. Participating in a wider range of activities is associated with higher well-being, as it allows for increased purpose through diverse roles in different social settings [31]. Empirically, this is supported by findings that older adults who were active more frequently, as measured by engagement in productive (i.e., volunteering/helping) and social (i.e., educational/athletic/religious/political) activities, had greater likelihood of having high well-being, as measured by life satisfaction, quality of life, self-rated health, psychological distress, chronic diseases, and body mass index [32]. These findings have been replicated internationally, suggesting a universal benefit to productive engagement in social activities [33]. In China, those who engaged in productive activities, such as work, caregiving, informal help, and volunteering, had slower decline in functional health [34].

Well-being, defined as optimal psychological functioning and experience [35], has also been linked to a wide range of health benefits [36]. Those with higher psychological and social well-being were shown to have a slower decline in physical functions, including walking speed, chair stands, and one-leg balance [26], while controlling for confounders by excluding those who had low physical functioning at baseline. The authors suggest that those with higher psychological well-being perceive their aging experience more positively, which encourages them to engage in preventive health behaviors, like eating healthy, exercising, and not smoking. Longitudinal studies have found that greater well-being is associated with lower cardiometabolic risk scores and lower risk of coronary heart disease 8 to 11 years later [37]. The association was found to be explained partially by health behaviors, such that people who were happier had better health behaviors, which translated into reduced risk of heart disease and better cardiometabolic risk levels. Similarly, those with stable high well-being over time reported better self-rated health, fewer health conditions, and less disability about 10 years later than those with stable low or stable moderate well-being over time, while controlling for earlier health characteristics [38].

Age-integrated networks might afford older adults unique well-being and social engagement benefits because the varied interactions tap into different mechanisms through which social connections benefit older adults. For example, intergenerational connections allow older adults to express generativity, or care and concern toward others [39], which has been linked to well-being and reduced mortality over time [40,41]. In addition, there are practical benefits of intergenerational connections, like learning about technology that can broaden older adults’ abilities to participate in activities and connect with others [12]. Connections with same-aged peers allow older adults to bond over shared experiences, encourage positive health-related behaviors (e.g., exercising together, eating healthy foods, not smoking), and relate to one another’s shared experiences, reinforcing social roles that make older adults feel like they are valued and belong [4].

### 1.2. Age Integration and Psychosocial Outcomes

The few studies that have examined the age composition of older adults’ social networks in relation to psychosocial outcomes vary in how they operationalize age integration. In a sample of European older adults, age integration was defined as upward if participants’ social networks included at least one non-kin social network member 10 years older than them, or downward if participants’ social networks included at least one non-kin social network member 10 years younger than them [11]. Another analysis operationalized older adults’ networks as age-integrated if they had two or more friendships with non-kin younger than 30 years old [10]. A final study used multivariate analyses to determine the sociodemographic predictors of having at least one non-kin member at least five years younger, and of having larger age gaps from one’s younger non-kin member [12].

Although these studies made valuable contributions to our understanding of older adults’ social networks, they also underscore the lack of consensus within and across studies on how to operationalize the construct of age integration and the lack of consideration for same-age peers as a feature of age-integrated social networks. Defining age integration as social connections with individuals in younger generations only does not reflect the greater level of age integration afforded to older adults connected with both same-aged peers and intergenerational ties. In order to understand age integration in social networks, it is necessary to define and measure age integration by considering intergenerational connections in the context of one’s social network, which may or may not also include same-age peers. The current study fills this gap in research by redefining and operationalizing age integration status of older adults’ social networks as age-integrated, peer-only, or intergenerational-only. By using a more comprehensive measure of age integration status, the current study is able to test the hypotheses that those with age-integrated networks have greater well-being and social engagement than those with peer-only and intergenerational-only networks. Sociodemographic features, including age, gender, race, education, and health, were also considered in the current study, as they have been linked to well-being [23,42,43,44], social engagement [45,46], and social network features [8,13,22,47,48].

## 2. Materials and Methods

### 2.1. Participants and Procedure

The National Health and Aging Trends Study (NHATS) was conducted to assess socio-economic factors associated with health and aging in a nationally representative sample of older adult Medicare beneficiaries (https://www.nhats.org). NHATS began in 2011 with data collection via in-person interviews with a nationally representative group of Medicare beneficiaries aged 65 or older. To correct for non-response biases, older and Black individuals were oversampled. Annual interviews were used to track changes in economic and social features over time. To maintain sufficient sample size, new participants were added in the fifth round of data collection.

The current study utilized data from the seventh wave of NHATS, conducted in 2016. Participants were excluded if they had no social network (*n* = 222), no social network data (*n* = 311), missing age information for all social network members (*n* = 1146), missing data on covariates (*n =* 595), and or/missing data on independent variables (*n* = 437). This resulted in a sample of 3564 participants, ranging in age from 67 to 101. Logistic regression analyses were conducted to determine whether those excluded from the analytic sample differed meaningfully from those included in the analytic sample. Participants who had higher well-being, more social activities, and who received help with self-care activities were less likely to be excluded from the sample. Conversely, women and Black, non-Hispanic participants were more likely to be excluded from the sample than men and White non-Hispanic participants, respectively. A priori power analyses conducted using G*Power determined the sufficient sample sizes to detect significant effects with power of 0.95, alpha of 0.05, and seven predictor variables in the well-being and social engagement ordinary least squares regression models were 141 and 120 participants, respectively.

### 2.2. Study Measures

Independent variable. Age integration status was derived using information about participants’ social networks. Specifically, participants were asked to name up to five individuals with whom they share important things in their lives (i.e., good or bad things that happen to [them], problems [they] are having, or important concerns [they] may have) to indicate social network membership. Social network members were excluded from the participants’ social network if their age was not reported by the participant. Based on prior research suggesting that a generation spans about 25 years [49], social network members were designated peers if they were within 25 years of the participants’ age, and intergenerational if they were at least 25 years younger than participants. Age integration status was considered peer-only if participants had only peers, intergenerational-only if they had only intergenerational connections, or age-integrated if they had both peers and intergenerational connections in their social networks.

Dependent variables. Well-being was calculated by summing the scores of 8 items measuring two dimensions of well-being, with items reverse scored such that higher scores indicated greater well-being (α = 0.72). The first subscale consisted of four items measuring hedonic, or emotional, well-being was endorsed on the following 5-point Likert scale: 1 = Never, 2 = Rarely (Once a week or less), 3 = Some days (2–4 days a week), 4 = Most days (5–6 days a week), 5 = Every day (7 days a week). Participants were asked how often, during the last month they felt cheerful, bored, full of life, and upset. The second subscale measuring eudaimonic, or psychological, well-being was assessed with 4 items, endorsed on a three-point Likert scale: 1 = Agree not at all, 2 = Agree a little, 3 = Agree a lot. Participants were asked how much they agree with statements including, “My life has meaning and purpose”, “I feel confident and good about myself”, “I gave up trying to improve my life a long time ago”, and “I like my living situation very much”. Social engagement was measured by counting the number of social activities older adults participated in during the previous month. Specifically, older adults were asked whether they participated in the following activities: (1) visit in person with friends or family; (2) attend religious services; (3) participate in clubs, classes, or other organized activities; (4) go out for enjoyment (e.g., going out to dinner, a movie, to gamble, or to hear music or see a play); (5) do volunteer work. Dummy codes for participation (coded as 1) or non-participation (coded as 0) in each activity were summed to indicate total number of social activities, with more social activities indicating greater social engagement.

Covariates. Demographic information included older adults’ age (in years), gender (male = 0, female = 1), and race (White, non-Hispanic = 0, Black, non-Hispanic = 1, Hispanic = 2, other [American Indian/Asian/Native Hawaiian] = 3). Sociodemographic information consisted of marital status (married/living with a partner = 0, divorced/separated = 1, widowed = 2, never married = 3) and education (high school or less = 0, more than high school = 1). Finally, health information included whether participants received self-care help (coded as 1) (e.g., help with bathing, dressing, etc.) in the past year, and number of health conditions, a count of chronic conditions participants reported (i.e., heart attack, stroke, etc.) ranging from 0–9.

### 2.3. Analysis Plan

All analyses were conducted using sample weights to account for non-response biases and to maintain sample representation of the Medicare population. First, descriptive statistics were conducted on the full sample. Then, bivariate descriptive statistics were stratified by age integration status to determine group differences. For categorical variables, chi-square analyses were conducted; for continuous variables, adjusted Wald tests were conducted. Next, we conducted separate ordinary least squares regression analyses for well-being and social engagement to determine their associations with age integration status and the covariates. All analyses were performed using Stata (Version 16.1, StataCorp LLC, College Station, TX, USA).

Supplementary analyses (Appendix A) demonstrated the confounding relationships between age integration status and marital status, parental status, and living arrangement. For example, widowed individuals rarely had peer-only networks because spouses who in most cases were same-aged peers were no longer in their social networks. On the other hand, married or partnered individuals rarely had intergenerational-only networks, because spouses/partners in most cases were same-aged peers. Therefore, these covariates were not included in the models. In addition, supplementary analyses in Appendix B demonstrate the statistically significant associations between the independent variable, covariates, and dependent variables to support the inclusion of covariates in the ordinary least squares regression analyses.

## 3. Results

### 3.1. Descriptive Analyses

Weighted descriptive statistics (Table 1) were conducted on the full sample (*n* = 3564). The average age of participants was almost 76 years old. The sample consisted of slightly more women (52.00%), White, non-Hispanic participants (83.38%), and participants with more than a high school education (62.10%). Most of the sample had peer-only networks (54.76%), almost a quarter had age-integrated networks, and 20.72% had intergenerational-only networks. Well-being ranged from 10 to 32, with an average of 26.97 for all participants. Social engagement ranged from 0 to 5, with participants averaging 3.02 social activities in the previous month.

Weighted descriptive statistics were stratified by age integration status to examine sociodemographic differences among groups (see Table 2). Chi-square analyses demonstrated significant associations between age integration status and gender (χ^2^ = 77.59, df = 2, *p* < 0.001) as well as race (χ^2^ = 30.78, df = 6, *p* < 0.05). There were no significant associations of age integration status with education (χ^2^ = 1.19, df = 2, *p* = 0.534) or self-care help (χ^2^= 4.04, df = 2, *p* = 0.145). Adjusted Wald tests revealed significant age differences among groups, F(2, 55) = 88.39, *p* < 0.001, with peer-only participants being the youngest (M = 74.50), age-integrated participants being slightly older (M = 76.60), and intergenerational-only participants being the oldest (M = 74.50). Age integration status was not significantly associated with number of health conditions, F(2, 55) = 2.86, *p* < 0.001.

Bivariate associations were also examined for outcome variables. Adjusted Wald tests revealed significant well-being differences among groups for social engagement only, F(2, 55) = 7.27, *p* < 0.01, with age-integrated participants having the greatest number of social activities and intergenerational-only participants having the fewest. Significant psychological well-being differences were not observed for participants with different age integration statuses, F(2, 55) = 2.56, *p* = 0.08.

### 3.2. Ordinary Least Squares Regression Analyses

Table 3 presents results from two ordinary least squares regression analyses conducted to determine the association between age integration status and well-being. The first model regressed age integration status on psychological well-being while controlling for covariates. When controlling for sociodemographic and health characteristics, age integration status was not associated with psychological well-being. Although the independent variable was not significantly associated with psychological well-being, several covariates were significant. Specifically, Black, non-Hispanic participants were predicted to have psychological well-being 0.81 units greater than their White, non-Hispanic counterparts; those with more than a high school education were predicted to have psychological well-being 0.72 units greater than those with a high school education or less; those who received self-care help were predicted to have psychological well-being 1.32 lower than those who did not receive self-care help; and those who had an additional health condition were predicted to have psychological well-being 0.41 lower.

In the second model, number of social activities was regressed on age integration status. Holding all other variables constant, age integration status was associated with social engagement, such that intergenerational-only participants participated in 0.30 fewer social activities than their age-integrated counterparts. In addition, women were predicted to participate in 0.32 more social activities than men, Hispanic and other-raced participants were predicted to participate in, respectively, 0.46 and 0.38 fewer social activities compared to White, non-Hispanic participants, those with more than a high school education were predicted to participate in 0.58 more social activities than those with a high school education or less, and those with self-care help and more chronic conditions were predicted to have 0.23 and 0.12 fewer social activities, respectively, controlling for all other variables.

## 4. Discussion

The present study contributes to our understanding of how older adults’ social networks relate to psychosocial outcomes. By characterizing the age integration status of older adults based on the presence of intergenerational-only connections, peer-only connections, or both, we expanded upon prior research that defined age integration as only having intergenerational connections. Given the well-documented associations of social integration (i.e., diverse social ties with varied social experiences) with mental well-being and social activity participation, we hypothesized that age-integrated social networks would similarly benefit older adults’ well-being and social engagement due to the diverse social connections experienced by those with both same-aged peers and intergenerational connections. This hypothesis was partially supported—age-integrated participants had significantly greater social engagement than those with intergenerational-only networks, but not those with peer-only networks; age integration status was not associated with well-being, which suggests that simply having a social convoy may be more important for psychological well-being than the age integration status of one’s social convoy.

Although the current study did not evaluate the mechanisms through which age-integrated networks increase social engagement, the hypothesis that having intergenerational and same-aged peer connections is associated with more social engagement than those with intergenerational-only networks was confirmed. Given that age-integrated networks inherently include different types of relationships, it is reasonable to assume that the different relationship types are associated with different social activities. For example, older adults may participate in clubs, classes, or organized activities where they engage with same-aged peers; or they may visit friends and family where they engage with intergenerational ties. Age-integrated individuals may participate in more social activities simply because their varied social connections afford them greater access to a range of social activities. Given that those with age-integrated networks had greater social engagement than those with intergenerational-only networks, but not those with peer-only networks, it can be deduced that same-aged peers provide more opportunities for social engagement than intergenerational connections alone. However, it is also possible that older adults with intergenerational ties resulting from intergenerational programs limit their engagement in other social activities compared to those with peer-only or age-integrated networks, reflecting a greater commitment to intergenerational programs. Although it is unclear the degree to which current data reflect the experiences of older adults who participate in formal intergenerational programs, the current study provides a basis for consideration of the full social convoy when evaluating benefits of intergenerational program participation. It is possible that intergenerational program participants with only intergenerational social ties do not benefit as much as those who also have same-aged peers for reasons suggested in this study, such that age-integrated older adults benefit from a wider variety of social engagement opportunities.

Social engagement was also related to sociodemographic features, including gender, race, education, and health. As previously documented [46], women participated in more social activities than men. Similarly, those with greater education participated in more social activities than their less-educated counterparts [46]. We also found that Hispanic and other-raced older adults were less socially engaged than White, non-Hispanic older adults, which may be related to cultural differences whereby Hispanic and Asian older adults’ collectivist cultures emphasize familial activities that may take the place of formal nonfamilial social participation [50]. Having more health conditions and receiving self-care help were also associated with fewer social activities, as health limitations have been posited to be both a precursor to and outcome of disengagement from social activities [18,51].

Well-being was not related to age integration status as hypothesized; however, several covariates, including race, education, and health were significantly related to well-being. Additional analyses investigated the potential moderating effects of race, education, and health in the association between age integration status and well-being, as these features have been linked to age integration status and well-being. None of the interactions was significant, suggesting that sociodemographic features are more strongly associated with well-being than age integration status. Specifically, Black, non-Hispanic participants had greater well-being that White, non-Hispanic participants. This finding has been replicated in other studies that have documented a racial paradox of well-being [44], as the cumulative disadvantage experienced by older Black individuals over their lifetime does not translate into having lower well-being or poorer mental health than older White individuals with more advantage. Additionally, replicated in other studies is the finding that more educated individuals have greater well-being [23]. This has been explained by their greater access to resources, social capital, and diverse social ties [52]. Finally, poorer health, as indicated by having more chronic conditions and especially having help with self-care activities, was associated with lower well-being, a finding that has also been demonstrated in previous research [53].

The findings of this study can inform our understanding of intergenerational programming benefits as the field continues to advance. It will be important to consider whether older adults who participate in intergenerational programs identify intergenerational connections in their social networks as a result of their intergenerational program participation, as this could reflect programmatic success. It is possible older adults’ intergenerational program participation may have an indirect effect on age integration status, such that even if they do not identify an intergenerational social tie from their program as a close social network member, they may be more likely to consider other intergenerational social ties in their lives as close social network members because of their positive intergenerational program experiences. For those who also have same-aged peer ties, older adult participants in intergenerational programs may benefit from the broader intergenerational ties directly or indirectly resulting from their program participation with greater social engagement, as suggested by the current study.

### Limitations and Future Directions

Although the current study advanced our understanding of the psychosocial benefits associated with age integration status, there are several limitations that should be noted. First, the cross-sectional nature of the data set precludes the causal nature of the relationship between age integration status and social activities. Although social networks may lead to better mental health and greater social engagement, it is also possible that better mental health and participation in more social activities determines social network features. It is likely that both statements are true to some extent: Schwartz and Litwin [24] showed that greater social connectedness over time was associated with better mental health over time, and at the same time, greater mental health over time was associated with greater social connectedness. They hypothesized that social networks would have a greater effect on later mental health; however, they found that mental health changes were more strongly associated with social networks, such that individuals who had greater well-being were encouraged to maintain and strengthen social ties. A longitudinal examination found that larger social networks were associated with greater engagement in social activities over a 6-year period [47]. The increased social stimulation and emotional support over time mediated the indirect relationship between larger social networks and improved well-being over time [47]. It is possible that participating in more social activities leads to richer social networks that include both same-aged peers and intergenerational connections. At the same time, it is possible that having both same-aged peers and intergenerational connections opens up opportunities to participate in more social activities with these social network members. Supplementary analyses in Appendix B support the possibility that greater social engagement predicts lower odds of having intergenerational-only compared to age-integrated networks; however, there remains no significant association between well-being and age integration status when reversing. Future studies can and should include additional NHATS data as it is released in order to evaluate the longitudinal associations of age integration status and psychosocial outcomes.

Second, participants were excluded if they did not provide ages for any of their social network members. This does not mean that those who were excluded from the analysis did not have social networks; rather, the lack of age information prevented us from determining whether social network members were intergenerational or same-aged peers. In some cases, participants remained in the sample because at least one of their social network members had age information, resulting in a less accurate representation of their complete social network. It is therefore possible that these social ties that could not be included in the present study have a significant influence on well-being and social engagement patterns that could not be captured. Although it is not known why age information was missing for some of the participants’ social network members, it is possible that these individuals were more peripheral social network members whose ages the participants simply did not know, as they might know their child’s or spouse’s ages. Social network literature suggests that peripheral, or weak, social ties are strong predictors of well-being and social engagement, as they provide opportunities for engagement in diverse settings [54,55]. In addition, we explored the use of multiple imputation to address missing demographic data and found that results were similar to those when using listwise deletion. Given the similar results and small amount of missing demographic data, we presented results with listwise deletion.

It should also be noted that prior studies assessing well-being and social engagement vary in operationalization of well-being and social engagement. For example, other studies consider absence of depressive symptoms or positive self-rated health to indicate psychological well-being [38,56,57]. The current study defines well-being as a distinct construct made up of eight items that capture subjective, psychological well-being better than simpler constructs available in NHATS, such as the two-item depressive symptoms assessment and one-item self-rated health assessment. In addition, the current study considered the quantity of social activities, but information on the frequency with which older adults participate in these activities was not available. It is possible that individuals who participate more frequently in fewer social activities may have greater well-being benefits than those who participate less frequently in more social activities, because they identify more strongly with their social roles, which provide them with more meaning and purpose than activities in which they are less committed [7,45,58]. It is also possible that another feature that has not been identified, such as one’s personality, is driving the association between age-integrated social networks and greater social engagement. For example, it may be that individuals with certain personality types (e.g., extroverted) are predisposed to having both age-integrated social networks and greater social engagement, in which case, the association between age integration status and social engagement is confounded. Similarly, it is possible that features of older adults’ environments that were not included in the analyses could influence age integration status, such as household type (e.g., assisted living facility, alone in the community, multigenerational household), or religious participation. Although we addressed the need to exclude confounding variables, including marital status, parental status, and living arrangement, it is important to consider the potential influences of these factors and religious participation on age integration status in future studies.

## 5. Conclusions

The current study provides new insight on social network benefits by distinguishing age-integrated networks from intergenerational-only and same-aged peer networks. Although age integration status was not associated with well-being benefits, it was associated with social engagement, with age-integrated individuals participating in more social activities than those with intergenerational-only networks. This demonstrates the value of distinguishing those with only intergenerational connections from those with intergenerational connections and same-aged peers. Future research should elucidate the causal nature of this relationship with longitudinal data. With the rise in intergenerational programs and increasing availability of opportunities for older adults to participate in intergenerational programs, it is possible that nationally representative data sets, like NHATS, can capture additional information on intergenerational program participation, and provide further support for psychosocial benefits of age-integrated networks. Additionally, evaluations of intergenerational programs might benefit from assessing the age integration status of older participants’ social networks to understand whether program outcomes vary for those with (i.e., age-integrated) and without same-aged peer ties (i.e., intergenerational-only).

## Figures and Tables

**Table 1:** Weighted Descriptive Statistics.

	All Participants *n* = 3564 *M* (*SE*) or %
***Dependent Variables***	
Well-Being (10–32)	26.97 (0.08)
Social Engagement (0–5)	3.02 (0.03)
***Independent Variable***	
Age Integration Status	
Peer	54.76
Intergenerational	20.72
Age-Integrated	24.52
***Covariates***	
Age (67–101)	75.95 (0.12)
Female	52.00
Race/Ethnicity	
White, non-Hispanic	83.38
Black, non-Hispanic	6.12
Hispanic	6.82
Other (American Indian/Asian/Native Hawaiian)	3.68
More than HS Education	62.10
Self-Care Help	16.14
Number of Health Conditions (0–9)	2.40 (0.03)

**Table 2:** Weighted Descriptive Statistics Stratified by Age Integration Status.

	Peer-Only *n* = 1768 *M* (*SE*) or %	Intergenerational-Only *n* = 890 *M* (*SE*) or %	Age-Integrated *n* = 906 *M* (*SE*) or %
***Dependent Variables***			
Well-Being (10–32)	27.09 (0.11)	26.65 (0.17)	26.99 (0.12)
Social Engagement (0–5)	3.03 (0.04)	2.85 (0.07)	3.15 (0.04)
***Covariates***			
Age (67–101)	74.50 (0.14)	79.03 (0.39)	76.60 (0.29)
Female *	46.22	65.03	53.89
Race/Ethnicity **			
White, non-Hispanic	85.23	77.16	84.50
Black, non-Hispanic	5.91	8.04	4.98
Hispanic	6.08	9.29	6.38
Other	2.78	5.51	4.15
More than HS Education	62.18	60.57	63.20
Self-Care Help	16.27	13.99	17.66
Number of Health Conditions (0–9)	2.35 (0.04)	2.52 (0.05)	2.39 (0.06)

Note. * *p* < 0.05, ** *p* < 0.01.

**Table 3:** Weighted Ordinary Least Squares Regression: Effect of Age Integration Status on Well-Being and Social Engagement.

	Well-Being Coefficient (*SE*)	Social Engagement Coefficient (*SE*)
***Independent Variable***		
Age Integration Status		
Peer-Only	0.07 (0.16)	−0.10 (0.06)
Intergenerational-Only	−0.33 (0.21)	−0.30 (0.07) ***
***Covariates***		
Age (67–101)	−0.01 (0.01)	0.00 (0.00)
Female	0.01 (0.14)	0.32 (0.05) ***
Race/Ethnicity		
Black, non-Hispanic	0.81 (0.17) ***	−0.06 (0.07)
Hispanic	0.48 (0.32)	−0.46 (0.11) ***
Other	0.54 (0.36)	−0.38 (0.15) *
More than HS Education	0.72 (0.15) ***	0.58 (0.05) ***
Self-Care Help	−1.32 (0.20) ***	−0.24 (0.07) **
Number of Health Conditions (0–9)	−0.41 (0.05) ***	−0.12 (0.02) ***

Note. Reference categories: age-integrated; male; White, non-Hispanic; high school education or less; no self-care help. * *p* < 0.05, ** *p* < 0.01, *** *p* < 0.001.

## Data Availability

The data presented in this study are available on request from the corresponding author. The data are not publicly available due to sensitive data.

## Appendix A

**Table A1:** Row Percentages, Age Integration Status and Confounding Variables.

	Peer-Only	Intergenerational-Only	Age-Integrated
***Marital Status***			
Married/Partnered	66.76	5.12	28.11
Divorced/Separated	39.64	47.95	12.41
Widowed	28.87	49.99	21.14
Never married	65.34	22.52	12.14
***Parental Status***			
Non-Parent	98.44	1.56	0.00
Parent	52.98	21.50	25.52
***Living Arrangement***			
Lives alone	30.61	49.57	19.82
Lives with spouse	67.45	4.80	27.75
Lives with others	66.00	5.67	28.33
Lives with spouse + others	38.01	44.81	17.19

## Appendix B

**Table A2:** Correlation Matrix of Dependent Variables, Independent Variable, and Covariates.

	Well-Being	Social Engagement	Age Integration Status	Age	Gender	Race	Education	Self-Care Help
***Dependent Variables***								
Well-Being	-							
Social Engagement	0.26	-						
***Independent Variable***								
Age Integration Status	−0.03	0.02	-					
***Covariates***								
Age	−0.10	−0.10	0.18	-				
Gender	−0.08	0.05	0.07	0.08	-			
Race	0.03	−0.14	0.00	−0.04	0.00	-		
Education	0.14	0.26	0.01	−0.14	−0.09	−0.16	-	
Self-Care Help	−0.19	−0.15	0.03	0.15	0.06	0.03	−0.07	-
Health Conditions	−0.22	−0.17	0.03	0.15	0.17	0.07	−0.14	0.28

## Appendix C

**Table A3:** Weighted Multinomial Logistic Regression, Well-Being and Social Engagement on Age Integration Status.

	Peer-Only *n =* 1768 *RRR [95% CI]*	Intergenerational-Only *n =* 890 *RRR [95% CI]*
***Independent Variable***		
Well-Being	1.01 [0.98, 1.04]	0.97 [0.93, 1.01]
***Covariates***		
Age (67–101)	0.95 [0.93, 0.96] ***	1.06 [1.04, 1.07] ***
Female *	0.73 [0.59, 0.89] **	1.60 [1.24, 2.05] ***
Race/Ethnicity		
Black, non-Hispanic	1.09 [0.81, 1.46]	2.02 [1.46, 2.80] ***
Hispanic	0.82 [0.52, 1.30]	1.89 [1.13, 3.16] *
Other	0.61 [0.32, 1.15]	1.63 [0.79, 3.34]
More than HS Education	0.82 [0.67, 1.02]	1.15 [0.90, 1.49]
Self-Care Help	0.98 [0.74, 1.29]	0.57 [0.41, 0.78] ***
Health Conditions	1.04 [0.96, 1.13]	1.01 [0.92, 1.10]
	**Peer-Only** ***n* = 1768** ***RRR [95% CI]***	**Intergenerational-Only** ***n* = 890** ***RRR [95% CI]***
***Independent Variable***		
Social Engagement	0.93 [0.86, 1.01]	0.81 [0.73, 0.89] ***
***Covariates***		
Age (67–101)	0.95 [0.93, 0.96] ***	1.06 [1.04, 1.07] ***
Female *	0.74 [0.60, 0.91] **	1.72 [1.33, 2.21] ***
Race/Ethnicity		
Black, non-Hispanic	1.09 [0.81, 1.46]	1.94 [1.40, 2.69] ***
Hispanic	0.80 [0.50, 1.27]	1.70 [1.01, 2.85] *
Other	0.60 [0.32, 1.12]	1.49 [0.72, 3.07]
More than HS Education	0.86 [0.69, 1.07]	1.28 [0.98, 1.66]
Self-Care Help	0.95 [0.72, 1.25]	0.55 [0.40, 0.75] ***
Health Conditions	1.03 [0.95, 1.11]	1.00 [0.92, 1.09]

Note. Reference categories: age-integrated; male; White, non-Hispanic; high school education or less; no self-care help. *RRR* = Relative Risk Ratio of having intergenerational-only or peer-only compared to age-integrated social networks. * *p* < 0.05, ** *p* < 0.01, *** *p* < 0.001.
